# Exploring Black Holes in Medical Education: Opportunities for Innovative Research

**DOI:** 10.12669/pjms.41.9.12435

**Published:** 2025-09

**Authors:** Ashfaq Akram

**Affiliations:** 1 Ashfaq Akram Department of Medical Education, Rai Medical College, Sargodha, Pakistan

In medical education, “Black Holes” are critical undeveloped areas that lack sufficient empirical evidence but carry significant consequences. Much like their astronomical counterparts, these gaps are often invisible, yet they distort key aspects of the educational universe: academic planning, student workload, program quality, and accreditation outcomes.

This communication identifies some major black holes (Fig.1) and explores why they matter as real-world challenges that impact learners and institutions alike. Greater attention, standardization, and targeted research are essential to bringing these hidden gaps into focus and strengthening the foundation of medical education.

**Fig.1:**
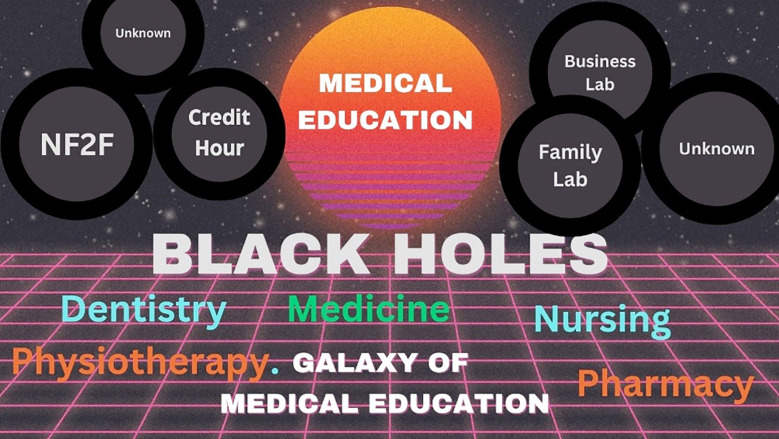
An Overview of Black Holes in the Galaxy of Health Education

Black Holes in Medical Education refers to hidden or overlooked aspects of teaching and learning that are not immediately visible but significantly influence the quality of education.

## Black Hole #1: Variability in Credit Hour Standards Across Global Medical Programs

Medical schools exhibit substantial variation in program length and credit hour allocation, reflecting differing educational philosophies, national regulations, and institutional goals. For instance, the MD program at Stanford University comprises 132 weeks of instruction^1^, whereas the University of Michigan requires 180 weeks. The Doctor of Medicine (MD) programs at the University of Sydney and the University of Melbourne are four-year graduate-entry courses. However, their credit hours calculation shows significantly different figures.

This discrepancy in credit allocation reflects institutional differences in curriculum design, workload measurement, and academic assessment frameworks. While both programs maintain rigorous standards and comprehensive clinical training, the variation in credit points does not necessarily indicate a difference in program intensity or content volume but rather highlights the distinct methodologies employed by each university to quantify academic engagement and learning outcomes. Similarly, King Saud University, the University of Sydney, and the United Arab Emirates University each follow unique program structures and time frames. While these programs are fully accredited and meet national standards, the lack of a universally accepted framework for defining credit hours presents a significant challenge.

A credit hour in medical education is typically determined based on a combination of instructional time and student workload. The American Medical Association (AMA) does not define credit hour calculations. The Liaison Committee on Medical Education (LCME) and the Accreditation Council for Graduate Medical Education (ACGME) set the credit hours.^2^,^3^ Generally, one credit hour represents approximately one hour of classroom instruction per week over a semester, supplemented by additional hours of out-of-class study, clinical experience, and practical training.^2^

In Europe, the European Association for Quality Assurance in Higher Education (ENQA) supports the use of the European Credit Transfer and Accumulation System (ECTS), where one credit hour typically equals 25-30 hours of total student workload, encompassing lectures, independent study time, and practical time.^4^ The UK medical programs follow the Credit Accumulation and Transfer Scheme (CATS), where one credit represents 10 hours of total student effort.^5^ Both systems emphasize a comprehensive measure of academic engagement rather than contact hours alone. The Australian Medical Council (AMC) adheres to the Australian Qualification Framework (AQF), where one credit point generally represents one hour of structured learning per week over a semester.^6^

Accrediting bodies such as the Liaison Committee on Medical Education (LCME), the Accreditation Council for Graduate Medical Education (ACGME), and the World Federation for Medical Education (WFME) offer general guidelines to support curriculum quality and consistency.^2^,^3^,^7^ However, they grant institutions considerable autonomy in determining how credit hours are calculated- particularly in balancing face-to-face instruction, clinical work, and self-directed learning. This flexibility leads to wide discrepancies in how credit hours are interpreted and assigned.

The absence of a standardized model complicates efforts to compare curricula across institutions and countries, undermining transparency in student workload expectations and making international equivalency assessments difficult. This inconsistency constitutes a critical yet often overlooked gap in medical education – what may be termed a Black Hole-that demands attention from policymakers, educators, and accreditation authorities alike. Establishing transparent and research-informed standards could enhance curriculum coherence, promote fairness, and improve the global portability of medical qualifications.

## Black Hole #2: The Grey Zone of Medical Education: Standardizing Non-Face-to-Face (NF2F) Student Learning Hours

While Face-to-Face (F2F) instructional time- such as lectures, laboratory sessions, and clinical rotations -is typically well-defined in medical curricula, Non-Face-to-Face (NF2F) learning time remains largely unquantified and inconsistently addressed.^8^ NF2F learning encompasses various student activities, including independent study, reading, assignments, literature review, and clinical skill preparation. Despite its critical role in shaping learning outcomes, this dimension of student engagement often lacks standardized measurement or documentation.

Akram et al.^9^ calculated that the NF2F study time in pharmacology was three to five hours, depending on content complexity and student attitude. Such variability underscores the absence of clear institutional guidelines and reveals the subjective nature of student workload estimation across different disciplines. Although studies have compared F2F and Online teaching across various medical and health-related disciplines, there remains a notable lack of literature addressing NF2F learning time. This gap highlights a critical need for empirical investigations that examine NF2F learning time as a distinct and measurable component within the broader educational framework.

In the U.S., the Fair Labor Standards Act (FLSA)^10^ defines a full-time employee as someone working at least 40 hours per week, usually spread over five days with 8-hour shifts. In the European Union, full-time workweeks are generally shorter, typically ranging from 35 to 37.5 hours. In medical education, accurately calculating NF2F time alongside F2F learning time is essential for determining the total Students’ Learning Time (SLT) and credit hours. It is still unclear whether a full-time student’s status should be a framework of 25, 30, or 40 hours.

The lack of a standardized, evidence-based definition of full-time student status-whether framed as 25, 30, or 40 hours per week -constitutes a significant and unresolved black hole in medical education. This ongoing uncertainty at the intersection of regulatory oversight and academic policy undermines consistency, academic integrity, and student well-being within structured educational frameworks. Regulatory authorities and accreditation bodies must collaboratively prioritize this issue, as addressing it through empirical research could open new avenues for inquiry and inform the development of coherent, evidence-informed policies across the medical education system.

The absence of empirical data and standardized benchmarks for NF2F learning time creates challenges in accurately calculating total SLT and assigning credit hours. It may lead to misaligned academic expectations, unbalanced workloads, and increased cognitive strain. Addressing this gap through systemic research and policy development is essential for improving curriculum transparency, promoting equity in workload distribution, and ensuring the integrity of credit hour assignments in medical education.

The NF2F learning varies considerably between preclinical and clinical disciplines, yet this variation is over-estimated in curriculum planning. During preclinical years, the subjects of anatomy, physiology, and biochemistry present differing levels of cognitive load and require varied study approaches. Anatomy requires extensive visual spatial memorization and repetition, while Physiology demands a conceptual understanding of dynamic body systems, and Biochemistry focuses on integrating molecular-level processes. Despite these differences, current curricula often apply uniform expectations for NF2F study time, failing to reflect the distinct learning strategies each subject necessitates.

The absence of tailored NF2F workload allocation risks undermining educational equity and efficiency. Recognizing and addressing these differences is essential for accurately estimating total SLT, improving instructional design, and ensuring fair credit hours assignments. Empirical research in this area is still limited, highlighting the need for focused investigations into how various disciplines influence independent learning requirements.

## Black Hole #3: The Unstructured Hour: Rethinking Teaching Schedules in Medical Education

Many medical universities have rigorous schedules with continuous teaching from 8 am to 4 pm. While intended to maximize instruction, such long periods without breaks can lead to student fatigue, reduced attention, and poor information retention. This cognitive overload may hinder effective learning, highlighting this area as an open call and a profound necessity for research in medical education.

Additionally, there is a notable lack of research on topic variation across academic disciplines. The educationists have not organized module content according to difficulty levels, resulting in the absence of definite guidelines within academic schedules. As a result, topics are scheduled for teaching without considering their level of complexity or priority, leading to a lack of structure and consistency in the academic timetable. However, there are no established rules for academic scheduling, which unlock new avenues for research.

The critical ‘Black Holes’ in medical education remain inquietly addressed in academic planning and policy. This editorial highlights key underexplored areas, such as NF2F learning time, inconsistencies in credit hour allocation, a clear definition of full-time academic engagement, and ineffective scheduling practices. These gaps affect the quality and equity of medical education and hinder evidence-based curriculum development. Addressing them requires a coordinated effort among educators, researchers, and accreditation bodies. Once thoroughly investigated and integrated into curriculum design, medical education will ensure transparency, fairness, and improved learning outcomes. Educators are encouraged to recognize the significance of these overlooked dimensions and prioritize them in future research and planning to shape a more coherent and effective educational landscape.
